# COVID-19 Vaccines and Thrombosis—Roadblock or Dead-End Street?

**DOI:** 10.3390/biom11071020

**Published:** 2021-07-13

**Authors:** Kenneth Lundstrom, Debmalya Barh, Bruce D. Uhal, Kazuo Takayama, Alaa A. A. Aljabali, Tarek Mohamed Abd El-Aziz, Amos Lal, Elrashdy M. Redwan, Parise Adadi, Gaurav Chauhan, Samendra P. Sherchan, Gajendra Kumar Azad, Nima Rezaei, Ángel Serrano-Aroca, Nicolas G. Bazan, Sk Sarif Hassan, Pritam Kumar Panda, Pabitra Pal Choudhury, Damiano Pizzol, Ramesh Kandimalla, Wagner Baetas-da-Cruz, Yogendra Kumar Mishra, Giorgio Palu, Adam M. Brufsky, Murtaza M. Tambuwala, Vladimir N. Uversky

**Affiliations:** 1 PanTherapeutics, CH 1095 Lutry, Switzerland; 2 Centre for Genomics and Applied Gene Technology, Institute of Integrative Omics and Applied Biotechnology (IIOAB), Purba Medinipur 721172, India; 3 Departamento de Genética, Ecologia e Evolução, Instituto de Ciências Biológicas, Universidade Federal de Minas Gerais, Belo Horizonte 31270-901, Brazil; 4 Department of Physiology, Michigan State University, East Lansing, MI 48824, USA; bduhal@gmail.com; 5 Center for iPS Cell Research and Application, Kyoto University, Kyoto 606-8397, Japan; kazuo.takayama@cira.kyoto-u.ac.jp; 6 Department of Pharmaceutics and Pharmaceutical Technology, Faculty of Pharmacy, Yarmouk University, P.O. Box 566, Irbid 21163, Jordan; alaaj@yu.edu.jo; 7 Zoology Department, Faculty of Science, Minia University, El-Minia 61519, Egypt; mohamedt1@uthscsa.edu; 8 Department of Cellular and Integrative Physiology, University of Texas Health Science Center at San Antonio, San Antonio, TX 78229, USA; 9 Department of Medicine, Division of Pulmonary and Critical Care Medicine, Mayo Clinic, Rochester, MN 55902, USA; manavamos@gmail.com; 10 Biological Science Department, Faculty of Science, King Abdulaziz University, Jeddah 21589, Saudi Arabia; lradwan@kau.edu.sa; 11 Department of Food Science, University of Otago, Dunedin 9054, New Zealand; pariseadadi@gmail.com; 12 School of Engineering and Sciences, Tecnológico de Monterrey, Av. Eugenio Garza Sada 2501 Sur, Monterrey 64849, Mexico; gchauhan@tec.mx; 13 Department of Environmental Health Sciences, Tulane University, New Orleans, LA 70112, USA; sshercha@tulane.edu; 14 Department of Zoology, Patna University, Patna 800005, India; gkazad@patnauniversity.ac.in; 15 Research Center for Immunodeficiency, Children’s Medical Center, Tehran University of Medical Sciences, Tehran 1416753955, Iran; rezaei_nima@yahoo.com; 16 Network of Immunity in Infection, Malignancy and Autoimmunity (NIIMA), Universal Scientific Education and Research Network (USERN), 17177 Stockholm, Sweden; 17 Biomaterials and Bioengineering Lab, Centro de Investigación Traslacional San Alberto Magno, Universidad Católica de Valencia San Vicente Mártir, c/Guillem de Castro 94, 46001 Valencia, Spain; angel.serrano@ucv.es; 18 Neuroscience Center of Excellence, School of Medicine, LSU Health New Orleans, New Orleans, LA 70112, USA; NBazan@lsuhsc.edu; 19 Department of Mathematics, Pingla Thana Mahavidyalaya, Maligram 721140, India; sarimif@gmail.com; 20 Condensed Matter Theory Group, Materials Theory Division, Department of Physics and Astronomy, Uppsala University, Box 516, 75120 Uppsala, Sweden; pritam.panda@physics.uu.se; 21 Applied Statistics Unit, Indian Statistical Institute, Kolkata 700108, India; pabitrapalchoudhury@gmail.com; 22 Italian Agency for Development Cooperation—Khartoum, Sudan Street 33, Al Amarat 11111, Sudan; damianopizzol8@gmail.com; 23 Applied Biology, CSIR-Indian Institute of Technology, Uppal Road, Tarnaka, Hyderabad 500007, India; ramesh.kandimalla@gmail.com; 24 Department of Biochemistry, Kakatiya Medical College, Warangal 506007, India; 25 Translational Laboratory in Molecular Physiology, Centre for Experimental Surgery, College of Medicine, Federal University of Rio de Janeiro (UFRJ), Rio de Janeiro 21941-901, Brazil; wagner.baetas@gmail.com; 26 Mads Clausen Institute, University of Southern Denmark, NanoSYD, Alsion 2, 6400 Sønderborg, Denmark; mishra@mci.sdu.dk; 27 Department of Molecular Medicine, University of Padova, 35122 Padova, PD, Italy; giorgio.palu@unipd.it; 28 UPMC Hillman Cancer Center, Division of Hematology/Oncology, Department of Medicine, University of Pittsburgh School of Medicine, Pittsburgh, PA 15213, USA; brufskyam@upmc.edu; 29 School of Pharmacy and Pharmaceutical Science, Ulster University, Coleraine BT52 1SA, UK; 30 Department of Molecular Medicine and USF Health Byrd Alzheimer’s Institute, Morsani College of Medicine, University of South Florida, Tampa, FL 33612, USA

**Keywords:** COVID-19, vaccines, SARS-CoV-2, thrombosis, chronic smokers

## Abstract

Two adenovirus-based vaccines, ChAdOx1 nCoV-19 and Ad26.COV2.S, and two mRNA-based vaccines, BNT162b2 and mRNA.1273, have been approved by the European Medicines Agency (EMA), and are invaluable in preventing and reducing the incidence of coronavirus disease-2019 (COVID-19). Recent reports have pointed to thrombosis with associated thrombocytopenia as an adverse effect occurring at a low frequency in some individuals after vaccination. The causes of such events may be related to SARS-CoV-2 spike protein interactions with different C-type lectin receptors, heparan sulfate proteoglycans (HSPGs) and the CD147 receptor, or to different soluble splice variants of the spike protein, adenovirus vector interactions with the CD46 receptor or platelet factor 4 antibodies. Similar findings have been reported for several viral diseases after vaccine administration. In addition, immunological mechanisms elicited by viral vectors related to cellular delivery could play a relevant role in individuals with certain genetic backgrounds. Although rare, the potential COVID-19 vaccine-induced immune thrombotic thrombocytopenia (VITT) requires immediate validation, especially in risk groups, such as the elderly, chronic smokers, and individuals with pre-existing incidences of thrombocytopenia; and if necessary, a reformulation of existing vaccines.

## 1. Introduction

The unprecedented development of several vaccines against coronavirus disease-2019 (COVID-19) promised that after 18 months of illnesses, deaths, confinements, and lockdowns, there was finally light at the end of the tunnel. Currently, four vaccines have been approved by the European Medicines Agency (EMA) that demonstrate protection against severe acute respiratory syndrome-Coronavirus-2 (SARS-CoV-2) variants, albeit with variable efficacy [1,2,3,4]. Notably, the lipid nanoparticle (LNP)-formulated mRNA COVID-19 vaccines BNT162b2 (Pfizer/BioNTech) [1] and mRNA-1273 (Moderna) [2] as well as the adenovirus (Ad)-based vaccines ChAdOx1 nCoV-19 (University of Oxford/AstraZeneca) [3] and Ad26.COV2.S (Johnson & Johnson/Janssen) [4]. Then, potentially more transmissible, and pathogenic variants, such as the B.1.1.7 UK variant [5] and the South African B.1.351 variant [6], were detected and shown to spread rapidly in different parts of the world. Preliminary data indicated that the B.1.1.7 variant provided an increased infection but not viral burden [7]. However, a recent study showed that individuals who tested positive for the B.1.1.7 variant had a 10-fold higher viral load than non-B.1.1.7 subjects [8]. A significant immediate concern was also whether current vaccines could provide protection against these new variants and other variants expected to emerge in the future. In the context of the BNT162b2 vaccine, the B.1.1.7 and B.1.351 variants showed antibody resistance [9]. Moreover, the ChAdOx1 nCoV-19 vaccine failed to provide protection against the B.1.351 variant in a clinical trial in South Africa [10]. These findings fostered the need for developing second-generation vaccines, capable of adjustment to the viral evolutionary variability and showing efficacy against newly emerged SARS-CoV-2 variants. As if that had not been bad enough, rare cases of thrombotic thrombocytopenia were then reported after vaccinations with the simian adenovirus AdChOx1 nCoV-19 vaccine [11,12]. In one study, 11 patients developed one or several thrombotic events 5–16 days after vaccination [12]. Nine patients had cerebral venous thrombosis, three had splanchnic-vein thrombosis, three had pulmonary embolism and four had other types of thromboses. Six patients died and five had disseminated intravascular coagulation. Cases of thrombosis associated with severe thrombocytopenia have also been reported after vaccinations with the Ad26.COV2.S vaccine [13]. Very recently, three cases of VITT were detected in females aged 44, 47 and 50 years at 7–12 days after the first vaccination with ChAdOx1 nCoV-19 and Ad26.COV2.S vaccines [14]. Additionally, thrombocytopenia has been reported in 20 individuals receiving RNA-based COVID-19 vaccines, 9 vaccinated with BNT162b2 (Pfizer/BioNTech) and 11 with mRNA-1273 (Moderna) [15].

## 2. Features of COVID-19 Vaccines and Thrombocytopenia 

All four COVID-19 vaccines mentioned earlier express the full-length SARS-CoV-2 S protein. It is expected that, being translated within the host cells, the S protein will be introduced to the immune system of the vaccinated patients as an antigen, which will elicit humoral and cellular immune responses providing protection for immunized individuals against SARS-CoV-2 infection [1,2,3,4]. Due to the recent discovery of rare cases of vaccine-induced thrombotic thrombocytopenia (VITT) it is important to analyze all vaccine components which might be associated with these events.

### 2.1. Tissue Plasminogen Activator (tPA) Leader Sequence and Thrombocytopenia Risk

The ChAdOx1 nCoV-19 vaccine is composed of the replication-deficient simian Ad vector ChAdOx1, expressing the full-length SARS-CoV-2 structural surface spike (S) glycoprotein gene downstream of the tissue plasminogen activator (tPA) leader or signal sequence [9]. The other Ad vector-based vaccine, Ad26.CoV2.S, also contains a tPA leader sequence, but additionally a stabilized SARS-CoV-2 S protein with a mutated furin site, and two consecutive prolines (PP) in the hinge region of S2 [16]. The tPA leader sequence is neither present in the BNT162b2 vaccine [1] nor in the mRNA-1273 vaccine [2].

**Figure 1:**
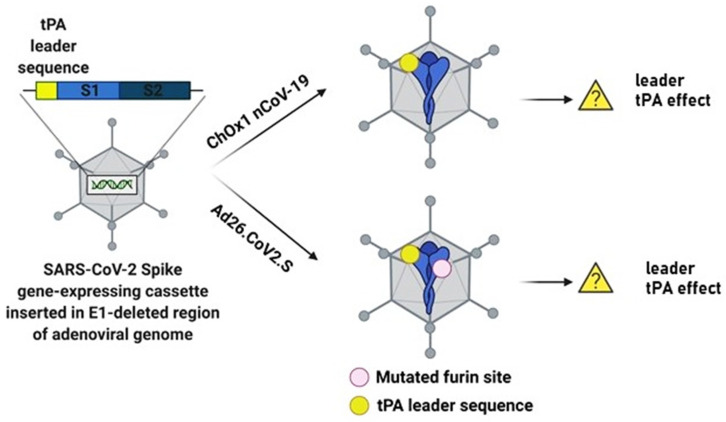
Schematic illustration of ChAdOx1 nCoV-19 and Ad26.COV2.S constructs and the potential association of tPA and thrombocytopenia. Both ChAdOx nCoV-19 and Ad26.COV2.S contain the tPA leader sequence and the full-length SARS-CoV-2 protein. Ad26.COV2.S additionally has a mutated furin site. The translation of only the tPA leader sequence is unlikely to cause thrombocytopenia as previously reported in ischemic stroke and acute myocardial infarction patients treated with recombinant tPA. Figure was created with Biorender.

Cases of thrombocytopenia have previously been reported in ischemic stroke and acute myocardial infarction patients after treatment with recombinant tPA [17,18,19,20], with 3.7% thrombocytopenia cases in 101,527 acute stroke patients treated with intravenous rtPA [20]. Therefore, the question has been raised whether the tPA leader sequence in the SARS-CoV-2 S protein expressed from the ChAdOx1 and Ad26 vectors, will have a similar effect in vaccinated individuals (Figure 1). However, the vaccination of 5 million individuals with the ChAdOx1 nCoV-19 vaccine in the European Economic area showed 30 rare cases of thromboembolic events, which was no higher than the number seen in the general population [21]. For this reason, the incidence of thrombotic thrombocytopenia related to the ChAdOx1 nCoV-19 and Ad26.COV2.S vaccine-derived rtPA is unlikely, as only the rtPA leader sequence is present in the vaccine vector.

### 2.2. Adenovirus-Induced Thrombocytopenia 

Adenoviruses (Ads) can naturally induce thrombocytopenia at a low frequency [22]. For instance, a 3-day old patient neonatally infected with Ad 40/41 developed thrombocytopenia [22]. Moreover, administration of an Ad5 vector in a mouse model was associated with the induction of thrombocytopenia 5–24 h after vector delivery [23]. The Ad vector transfer was associated with increased platelet and leukocyte-derived microparticles and multimers of the von Willebrand factor. Moreover, the fiber protein in Ad has been associated with thrombocytopenia, and the fiber protein of Ad5 in particular can trigger cytokine activation leading to Ad-induced thrombocytopenia [24]. Ad vectors have also caused dose-dependent thrombocytopenia in rhesus macaques by increasing in vivo platelet clearance [25]. 

In the case of Ad-based vaccine applications, two phase II clinical trials have been conducted for Ebola virus vaccines [26,27]. The simian ChAdOx1 vector expressing the Ebola virus glycoprotein (ChAd3-EBO-Z) was administered to 1509 adults, and although injection site pain and serious non-vaccine related events were recorded, no clinically meaningful thrombocytopenia was registered [26]. In the other phase II trial, the ChAd3-EBO-Z vaccine was administered to 300 healthy children, six years old or younger [27]. Common injection site pain and fever were observed and two serious adverse events unrelated to the vaccine were registered. The combined number of patients vaccinated with the ChAd3-EBO-Z in the phase I and II trials did not exceed 2000, which makes is unlikely that rare cases of thrombocytopenia could be discovered.

Although the majority of the vaccine-associated thrombosis and thrombocytopenia cases have been described for the ChAdOx1 nCoV-19 vaccine [11], cases have also been reported from vaccinations with the Ad26.COV2.S vaccine [13]. A case of extensive thrombosis associated with severe thrombocytopenia, which resembled autoimmune heparin-induced thrombocytopenia [28], was described for a patient vaccinated with the Ad26.COV2.S vaccine [13]. It is important to point out that the human Ad26 and the simian ChAdOx1 vectors use different entry receptors [29]; Ad26 interacts with the cellular receptor CD46, and ChAdOx1 mainly binds to the Coxsackie and adenovirus receptor (CAR) [29]. The binding of the Ad26 vector to the CD46 receptor could trigger upregulation of the complement pathways leading to thrombosis events [30]. The ChAdOx1 vector can potentially interact with human platelets, as CARs have been detected on these cells [31].

## 3. Structural and Biological Features of the Spike Protein and Thrombosis Risk 

Although the ACE2 receptor is the primary entry receptor, multiple host cell components interact with the SARS-CoV-2 S protein, which facilitate viral entry and can contribute to the spread of the pandemic [32]. Heparan sulfate proteoglycans (HSPGs), C-type lectin receptors (CLRs), and extracellular matrix metalloproteinase (CD147) on the host cell surface are potential targets for the S protein [32,33]. In the case of both the current Ad- and mRNA-based COVID-19 vaccines, the SARS-CoV-2 S protein could potentially induce thrombotic thrombocytopenia. In this context, a case of thrombosis-related fatality was recently detected in a person receiving the BNT162b2 mRNA vaccine [34]. Additionally, thrombocytopenia has been reported in 20 individuals receiving RNA-based COVID-19 vaccines, 9 vaccinated with BNT162b2 (Pfizer/BioNTech) and 11 with mRNA-1273 (Moderna) [15]. 

**Figure 2:**
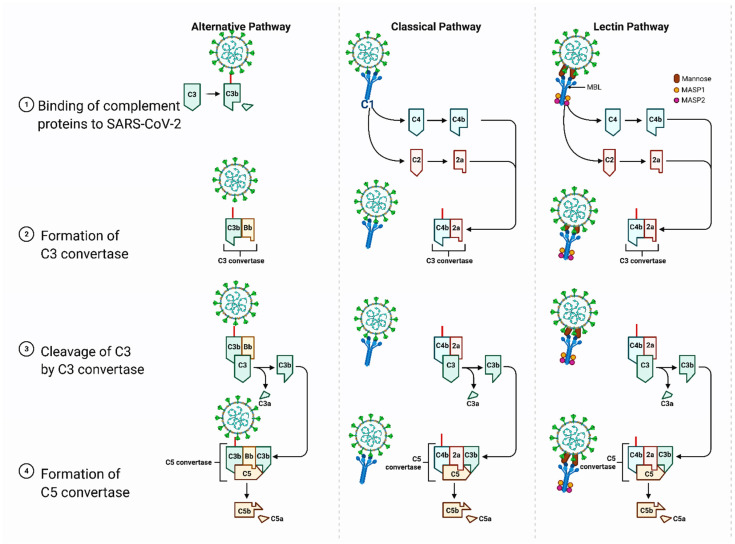
Potential Mechanisms for Upregulation of Complementary Pathways by the SARS-CoV-2 S Protein. Schematic presentation of the stages for alternative, classic and lectin pathways: **1**. Binding of complement proteins to SARS-CoV-2; **2**. Formation of C3 convertase; **3**. Cleavage of C3 by C3 convertase; **4**. Formation of C5 convertase. Figure was created with Biorender.

There are several potential mechanisms related to the upregulation of complement pathways by the SARS-CoV-2 S protein that could lead to thrombosis (Figure 2). For instance, an interaction of the S protein with the HSPGs on the cell membrane can interfere with the negative regulator of the complement alternative pathway, factor H protein, which leads to an inflammatory response through the downstream C3c convertase protein [35]. The flat sialic acid-binding domain of the SARS-CoV-2 S protein is likely to accelerate the movement of virions and increase the rate of detection by the mannose-binding lectin (MBL) of the complement lectin pathway, which has been associated with the fatality of COVID-19 patients [36]. Furthermore, the sialic acid-binding immunoglobulin-type lectins (SIGLECs), mainly SIGLEC7 and others, such as SIGLEC5, SIGLEC9, and SIGLEC11, have been associated with severe cases of COVID-19, suggesting the importance of these receptors that could be regulated by the enhanced sialic acid affinity of the S protein [32,37]. Therefore, the vaccines containing the full-length SARS-CoV-2 S protein could potentially trigger the complement pathway leading to thrombosis events. However, complement activation has only been seen in severe cases of COVID-19 so far [38]. 

An interesting hypothesis was recently described based on findings that the transcription of wildtype and codon-optimized SARS-CoV-2 S proteins enabled alternative splicing, which generated C-terminally truncated soluble S protein variants [39]. These variants might bind to ACE2 receptors on endothelial cells in blood vessels, triggering severe side effects similar to the thromboembolic events described by the SARS-CoV-2 encoded S protein. The underlying disease mechanism was termed Vaccine-Induced COVID-19 Mimicry (VIC19M) syndrome [39]. 

### 3.1. Heparin-Induced Thrombocytopenia and the Possible Role of Vaccines Containing the SARS-CoV-2 Spike Protein 

**Figure 3:**
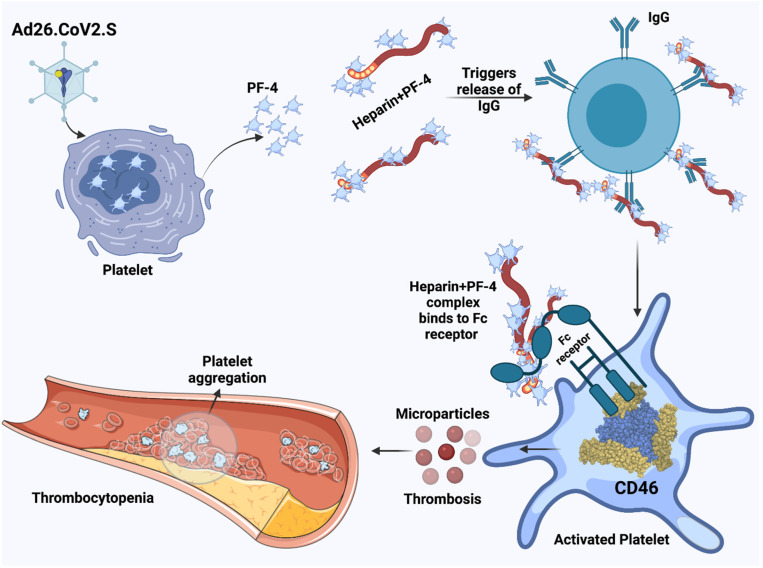
Heparin-induced Thrombocytopenia. Proposed mechanism of thrombocytopenia induced after Ad-based vaccine administration. Figure was created with Biorender.

It was recently demonstrated that in several cases of individuals with thrombotic events and thrombocytopenia after ChAdOx1 nCoV-91 vaccination, who elicited antibody responses against platelet factor 4 (PF4)-heparin, also generated antibodies against PF4 independent of heparin (Figure 3) [11]. However, the enhanced reactivity of patient sera with platelets could be an in vitro artifact due to the addition of the vaccine vector, as adenovirus has been demonstrated to bind to platelets causing their activation [40]. Additionally, the quantity of adenovirus in the 0.5 mL vaccine dose is unlikely to contribute to the platelet activation detected in these thrombocytopenia patients [11]. 

However, DNA released from the vaccine could potentially trigger PF4-reactive antibodies, as it has been previously shown that DNA and RNA can form multimolecular complexes with PF4 and lead to antibody binding in patients with heparin-induced thrombocytopenia and the induction of antibodies against PF4-heparin in a mouse model [41]. In another publication, venous thrombosis and thrombocytopenia were described in five healthcare workers 7–10 days after the first dose of ChAdOx1 nCoV-19 [12]. High levels of PF4-polyanion complexes were detected in all individuals, although they had not previously been exposed to heparin. It was suggested that these cases represent a rare vaccine-related variant of spontaneous heparin-induced thrombocytopenia [12]. In the context of the single-dose Ad26.COV2.S vaccine, there is a case report of a 48-year old white woman with an unremarkable medical history who developed extensive thrombosis which was associated with severe thrombocytopenia [13]. Furthermore, strong antibody responses against PF4-polyanion were demonstrated and the intravascular coagulation resembled autoimmune heparin-induced thrombocytopenia. Due to additional cases of rare and severe thrombocytopenia in individuals receiving the Ad26.COV2.S vaccine, the US Centers for Disease Control and Prevention (CDC) and the Food and Drug Administration (FDA) recommended pausing vaccinations until the cases had been reviewed [42]. In another report, after ChAdOx1 nCoV-19 vaccinations, 22 patients with acute thrombocytopenia and primarily cerebral venous sinus thrombosis (CVST) and one patient with isolated thrombocytopenia and a hemorrhagic phenotype were observed [43]. A similar blood clot type has been identified in a relatively small number of patients vaccinated with the ChAdOx1 nCoV-19 vaccine [11,12,43]. In the classic model of heparin-induced thrombocytopenia, the PF4 interaction with endothelial cell HSPGs leads to the removal of antithrombin that is connected to the HSPGs [44]. Additionally, antibodies to PF4/heparin bind and activate cellular FcγRIIA on platelets and monocytes, which lead to hypercoagulation and potentially life-threatening thrombosis [44]. As aforementioned, the S protein has a proposed capacity to interact with epithelial HSPGs, which could trigger the translocation of antithrombin, and heparin could activate the anti-PF4 antibodies resulting in heparin-induced thrombocytopenia. As negative PF4 cases of VITT are possible, it is essential to establish functional antibody testing to confirm VITT. However, many tests such as chemiluminescence immunoassay analyzer (CLIA) are useless when applied alone, but the combination of the sensitive ELISA-based PF4/heparin immunoassay together with a negative CLIA will allow identification of VITT antibodies [45]. In the context of the diagnostics of heparin-induced thrombocytopenia, the PF4-heparin ELISA lacks specificity, and the gold standard carbon 14-labeled serotonin release assay (SRA) has its limitations, so the PF4-dependent P-selectin expression assay (PEA) may be the solution for rapid and conclusive testing [46].

### 3.2. Potential Role of the CLR DC-SIGN in the Development of Thrombosis

Both SARS-CoV-2 and SARS-CoV target the CLR DC-SIGN (dendritic cell-specific ICAM-3 grabbing non-integrin 2) and CD209 antigen for host cell entry [32]. There is growing evidence suggesting the role of the CLRs CD209L and CD209 as entry receptors for SARS-CoV-2, especially in tissues with low ACE2 receptor presence or even with no ACE2 expression [47]. Importantly, it has previously been demonstrated that DC-SIGN and C-type lectin-like receptor 2 (CLEC2) can mediate human immunodeficiency virus type 1 (HIV-1) capture by platelets, contributing to thrombocytopenia [48]. Therefore, vaccine-mediated expression of the SARS-CoV-2 S protein might contribute to platelet-induced thrombocytopenia. However, the VITT relies on circulating S protein and not anti-S immune responses, which raises the question whether sufficient levels of secreted S protein will be available. 

### 3.3. Potential Role of the CD147 Receptor in the Development of Thrombosis

CD147 (also known as Basigin (BSG) or EMMPRIN) has also been suggested as another receptor for SARS-CoV-2 [33]. Previously, both SARS-CoV and HIV-1 have been reported to utilize CD147 as a ligand [33], but no direct role for CD147 in host cell entry of SARS-CoV-2 has been identified so far. Especially in older COVID-19 patients, who possess lower ACE2 but higher CD147 expression levels, CD147 has been associated with thrombosis [33] and could have a role in COVID-19 VITT.

## 4. Potential Risk Groups for Thrombosis 

The number of cases of VITT after the administration of both Ad- and mRNA-based COVID-19 vaccines is very low and there is broad consensus among experts that the benefits of vaccination are superior to the potential risks of severe vaccine effects such as VITT [49]. A meta-analysis of 27 studies in 3342 COVID-19 patients showed an incidence rate of 16.5% of pulmonary embolism (PE) and 14.8% of deep vein thrombosis (DVT) [50]. In contrast, 11 excess venous thromboembolic events per 100,000 vaccinations with the ChAdOx1 nCoV-19 vaccine were detected in Denmark and Norway [51]. However, no increase in the rate of overall arterial events were observed, but a slightly elevated rate of thrombocytopenia disorders and bleeding were recorded. 

Moreover, the VITT risk post-vaccination is much lower compared to, for instance, smoking and oral contraceptives. In comparison to never smokers, the venous thromboembolism (VTE) overall combined relative risk (RRs) for ever smokers was 1.17 (95% CI 1.09–1.25), for current smokers 1.23 (95% CI 1.14–1.33) and for former smokers 1.10 (95% CI 1.03–1.17) indicating a slightly increased risk of VTE in smokers [52]. As the average risk of VTE, DVT and PE in women of childbearing age has been estimated to 2–10 cases per 10,000 individuals per year, with the average odds of roughly 1:1700 [53], the use of contraceptives can increase the risk 3 to 6-fold [54,55]. Based on the estimated 3-fold increase the odds of developing VTE are 1:550, which is much higher than for the development of VTE in general (1:170,000) or VITT after COVD-19 vaccination (1:100,000) [18]. Interestingly, age seems to play a role in VTE/VITT cases, as a higher incidence rate of 18.0 per million doses has been reported in younger adults (18–49 years) compared to 12.0 per million doses in older individuals (>50 years) after the first dose of the ChAdOx1 nCoV-19 vaccine [56]. On the other hand, the overall VITT incidence after the second dose was 1.3 per million doses. So far, no cases have been detected in the 18–49 years group despite 2.7 million individuals having received both doses. Moreover, VITT cases were mainly reported in females.

## 5. Conclusions

This study provides a perspective on the potential mechanisms of VITT. As described, both Ad- and mRNA-based vaccines have been associated with induced thrombocytopenia, although at a very low frequency taking into account the extent of COVID-19 mass vaccinations. Different theories have been presented for thrombocytopenia development. Although ischemic stroke and acute myocardial infarction have been detected in patients treated with tPA, the presence of only the tPA leader sequence in the ChAdOx1 vector is highly unlikely to induce thrombocytopenia. A possibility comprises Ad itself inducing thrombocytopenia as has previously been described [19]. It relates to an Ad-induced increase in platelets, but also the cytokine activation triggered by the Ad fiber protein. However, thrombocytopenia has also been detected after vaccinations with mRNA-based COVID-19 vaccines. As both Ad- and mRNA-based vaccines use the full-length SARS-CoV-2 S protein as an antigen, its interaction with multiple membrane components might induce thrombocytopenia. Alternatively, heparin might activate anti-PF4 antibodies after ChAdOx1 nCoV-19 vaccination, resulting in heparin-induced thrombocytopenia. Other factors affecting thrombocytopenia consist of the C-type lectin receptor DC-SIGN and the CD147 receptor. 

Based on the above discussed pathogenic mechanisms, the issue of the management of acute and subacute/chronic forms of CSVT with VITT has been addressed [57]. It should, as for treatment of heparin-induced thrombocytopenia with thrombosis, basically include alternative anticoagulants to heparin or a direct oral anticoagulant (DOAC). 

Regardless of which vaccine is used, it is obvious that the risk of developing post-vaccination thrombocytopenia is much lower than the risk of death and morbidity from SARS-CoV-2 infections. Vaccines are vital and invaluable to control the COVID-19 pandemic and to build up herd immunity against SARS-CoV-2. For this reason, it is important to thoroughly investigate the reasons behind VITT and take the appropriate actions related to individuals with a pre-existing susceptibility to thrombocytopenia and if necessary, re-engineer both vaccine vectors and formulations to ensure that we have only encountered a roadblock and not reached a dead-end street.

## Data Availability

No new data were created or analyzed in this study. Data sharing is not applicable to this article.
